# Releasing fast and slow: Non-destructive prediction of density and drug release from SLS 3D printed tablets using NIR spectroscopy

**DOI:** 10.1016/j.ijpx.2022.100148

**Published:** 2022-12-17

**Authors:** Sarah J. Trenfield, Xiaoyan Xu, Alvaro Goyanes, Martin Rowland, David Wilsdon, Simon Gaisford, Abdul W. Basit

**Affiliations:** a UCL School of Pharmacy, University College London, 29-39 Brunswick Square, London WC1N 1AX, UK; b Departamento de Farmacología, Farmacia y Tecnología Farmacéutica, R + D Pharma Group (GI-1645), Universidade de Santiago de Compostela, 15782, Spain; c Pfizer Ltd., Drug Product Design, Discovery Park, Ramsgate Road, Sandwich CT13 9ND, UK; d Pfizer Ltd., 280 Shennecossett Road, Groton, CT 06340, United States

**Keywords:** 3D printed medicines, Additive manufacturing, Process analytical technology tools, Powder bed fusion printing, Printed drug delivery systems and pharmaceuticals

## Abstract

Selective laser sintering (SLS) 3D printing is a revolutionary 3D printing technology that has been found capable of creating drug products with varied release profiles by changing the laser scanning speed. Here, SLS 3D printed formulations (printlets) loaded with a narrow therapeutic index drug (theophylline) were produced using SLS 3D printing at varying laser scanning speeds (100–180 mm/s). The use of reflectance Fourier Transform – Near Infrared (FT-NIR) spectroscopy was evaluated as a non-destructive approach to predicting 3D printed tablet density and drug release at 2 h and 4 h. The printed drug products formulated with a higher laser speed exhibited an accelerated drug release and reduced density compared with the slower laser scanning speeds. Univariate calibration models were developed based on a baseline shift in the spectra in the third overtone region upon changing physical properties. For density prediction, the developed univariate model had high linearity (R^2^ value = 0.9335) and accuracy (error < 0.029 mg/mm^3^). For drug release prediction at 2 h and 4 h, the developed univariate models demonstrated a linear correlation (R^2^ values of 0.9383 and 0.9167, respectively) and accuracy (error < 4.4%). The predicted vs. actual dissolution profiles were found to be statistically similar (f_2_ > 50) for all of the test printlets. Overall, this article demonstrates the feasibility of SLS 3D printing to produce drug products containing a narrow therapeutic index drug across a range of drug release profiles, as well as the potential for FT-NIR spectroscopy to predict the physical characteristics of SLS 3D printed drug products (drug release and density) as a non-destructive quality control method at the point-of-care.

## Graphical abstract

**Figure:**
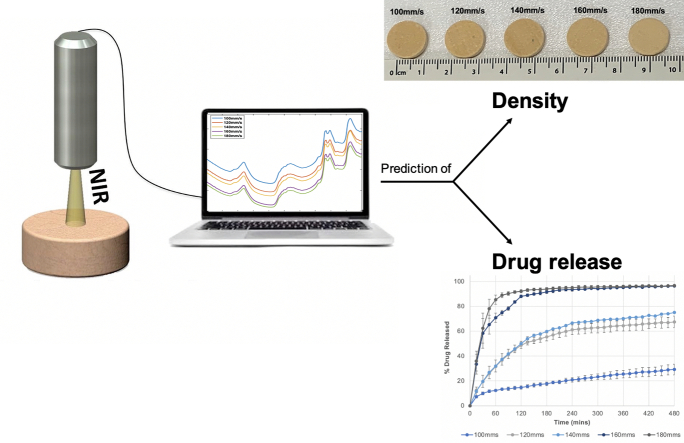

## 1 Introduction

Selective laser sintering (SLS) 3D printing technology is a revolutionary drug product manufacturing technology capable of producing medicines with personalised and flexible characteristics on demand (Awad et al., 2021; Seoane-Viaño et al., 2021). The technology uses a diode laser to cause a partial or full sintering (fusion of powder particles) of a drug-loaded powder-bed feedstock in a layer-by-layer process. Following each layer sintering, a roller distributes a fresh layer of powder on top of the sintered object, which is repeated to produce 3D-printed tablets (Printlets™). This 3D printing process has been widely researched to have numerous benefits within pharmaceuticals, enabling the production of drug products with personalised dosages (Gueche et al., 2021c; Kulinowski et al., 2022; Trenfield et al., 2018), shapes (Awad et al., 2020), sizes (Awad et al., 2019), solid state properties (Davis Jr. et al., 2021; Madžarević et al., 2021; Santitewagun et al., 2022; Thakkar et al., 2021; Trenfield et al., 2022), and multi-drug combinations (Trenfield et al., 2020).

Interestingly, previous studies have also shown that drug release from SLS 3D printed drug products can be controlled by varying the drug load (Fina et al., 2017), excipient type and load (Allahham et al., 2020; Gueche et al., 2021a), printlet geometry (Fina et al., 2018a) and laser scanning speed of the process (Barakh Ali et al., 2019; Fina et al., 2018b; Gueche et al., 2021b; Khuroo et al., 2022). Fina. et al. showed that slower laser scanning speeds produced drug products with an increased density and reduced porosity compared with their faster scanning speed counterparts (Fina et al., 2018b). This had a direct effect on the rate of drug dissolution, with faster laser scanning speeds causing an accelerated drug release for two different polymer types (HPMC and Kollicoat IR) (Fina et al., 2018b). This concept could have significant benefits for the pharmaceutical industry, enabling a tailored release profile to be produced without the need for the design of a new and distinct drug product composition.

Currently, dissolution is the key method in the pharmaceutical industry for evaluating drug release from solid oral dosage forms and this method can have some degree of relevance to in vivo *bioavailability and hence* therapeutic efficacy (Gray et al., 2009). However, dissolution testing involves a series of time-consuming, labour-intensive and expensive analytical tasks, involving protocols such as instrument calibration, media preparation, sample collection, drug assay and data analysis, as well as requiring large amounts of solvents for testing (Nagy et al., 2019). Furthermore, dissolution results can be highly variable, often originating from the drug product variability itself (e.g., drug or excipient grades, dosage form hardness and porosity) or the inherent variability associated with dissolution methodology (instrumentation, location of analysis, methods for sample withdrawal and analysis, buffer media factors) (Qureshi and Shabnam, 2001).

It is clear that these tests are impractical for the analysis of 3D printed medicines as they are inherently time consuming, costly, require trained personnel, require equipment and laboratory space and are highly destructive making it impossible to fit the ‘on-demand’ printing model, as well as challenging to identify the root of the problem if a test fails or if anomalies occur. One strategy could be to produce extra samples via 3D printing to undergo external quantity and quality analysis. However, it would not be economically feasible for the manufacturing of personalised medicines (Edinger et al., 2017). As such, there is a demand for the development of non-destructive and real-time methods to evaluate drug release and performance for these novel drug products.

Recent research has highlighted the potential for rapid and non-destructive spectroscopic methods to provide non-destructive quality control processes for 3D printed drug products. Our group has previously shown the potential for spectroscopic methods to successfully predict the dosage of a single and multiple drugs within 3D printed tablets (Trenfield et al., 2018; Trenfield et al., 2020), as well as to non-destructively predict the solid-state form of a BCS II drug (Trenfield et al., 2022). Studies have also shown the potential for near infrared (NIR) spectroscopy to predict physical characteristics of drug products, such as drug release and drug product porosity for matrix tablets. Tabasi. et al. used reflectance NIR spectroscopy in combination with PLS regression analyses to determine the relationship between drug release and polymer content (Tabasi et al., 2009). Their study involved the production of theophylline matrix tablets with differing polymer loads (different amounts of Eudragit NE 30D: 0% - 25% *w/w*). They found that an increase in polymer content caused an increase in NIR absorbance which could be correlated to drug release at 1 h, 2 h and 4 h. Previous studies have also utilised NIR spectroscopy for the prediction of drug dissolution upon changing drug load (Pawar et al., 2016), mixing time (Abe and Otsuka, 2012), strain (Hernandez et al., 2016) and compaction pressures (Blanco et al., 2006; Donoso and Ghaly, 2004). Whilst extensive research has been conducted for prediction of drug product density and dissolution using NIR spectroscopy for conventional compressed tablets, so far, no such research has been conducted for 3D printed formulations.

As such, the objective of this study was to firstly evaluate the impact of SLS laser scanning speed (100–180 mm/s) on density and drug release from matrix 3D printed tablets (printlets) loaded with a model narrow therapeutic index (NTI) drug; theophylline. The developed SLS printlets were scanned using reflectance Fourier Transform (FT)-NIR spectroscopy and subsequent univariate models were developed and evaluated for their abilities to predict drug product density and drug dissolution at 2 h and 4 h. The physical characteristics (tablet hardness, density and drug load), drug dissolution in a USP II apparatus, as well as solid state characteristics of the dosage forms were also evaluated.

## 2 Methods

### 2.1 Materials and methods

Theophylline anhydrous USP grade (Sigma-Aldrich, 2022, UK) was used as a model narrow therapeutic index drug (MW 180.16 g/mol, solubility in water: 8.3 mg/mL (Sigma-Aldrich), >99% purity). Eudragit L100–55, a copolymer of methacrylic acid and ethyl acrylate (1:1 ratio) that dissolves at pH 5.5 was donated by Evonik, UK. Candurin Gold Sheen was purchased from Merck, UK. The salts for preparing the buffer dissolution media were purchased from VWR International Ltd., UK.

### 2.2 Printing process

For each formulation 100 g of powder mixture was made by combining 10% theophylline, 87% Eudragit L100–55 and 3% Candurin Gold Sheen in a pestle and mortar. 3% of the colourant Candurin® Gold Sheen was added to each formulation as an absorbent, to enhance laser energy absorption and to ensure printability. The powder was then transferred to the SLS printer (Sintratec Kit, AG, Brugg, Switzerland) to formulate the 3D printed tablets (printlets). 123D Design (Autodesk, United States) was used to design the shape of the cylindrical tablets (15 mm diameter x 3.6 mm height). The designed 3D models were exported as a stereolithography (.stl) file into the 3D printer Sintratec central software Version 1.1.13.

The powder mixture was added to the building platform (130 × 130 × 30 mm) which was set in its highest position, where the blade was moved across to flatten and create an even and homogenous powder bed for printing. The chamber temperature (90 *°*C) and platform surface temperature (110 *°*C) were kept constant throughout the experiment. The laser speed was the only variable where the printlets were printed using 9 different laser speeds (100 mm/s, 110 mm/s, 120 mm/s, 130 mm/s, 140 mm/s, 150 mm/s, 160 mm/s, 170 mm/s and 180 mm/s, *n* = 5). Each set of formulations took between 5 and 8 min to print. The printing process began by the activation of a 2.3 W blue diode laser (445 nm) to sinter the first layer of powder onto the building platform, based on the pattern in the .stl file. As soon as the laser had stopped sintering the first layer, the roller distributed a new powder layer over the previously sintered area. This process was repeated layer-by-layer until the desired object was completed. Individual tablets were removed from the printer once cooled and any excess unsintered powder was brushed off. Three printlets of the same speed were printed at the same time.

### 2.3 Near infrared spectroscopy (NIR) data acquisition

Printlets were scanned on the integrating sphere using a small cup assembly in reflectance mode using a fourier transform (FT)-NIR spectrometer (MPA, Bruker, Germany). For reflectance measurements, spectra were collected twice on each side of each printlet across a wavenumber range of 12,800 to 4000 cm^−1^ and at a resolution of 8 cm^−1^ totaling 64 scans, which were averaged. Collection of the data was performed using OPUS Version 6.5 software (Bruker, United States).

### 2.4 Calibration model development

Univariate calibration models were developed by plotting the NIR absorbance at 9000 cm^−1^ against density and drug release at 2 h and 4 h using Microsoft Excel (v16.39). During univariate model development, all speeds were included in the calibration set and five printlets (covering 100 mm/s, 120 mm/s, 140 mm/s, 160 mm/s and 180 mm/s) were set aside and used as a test set to evaluate the predictive ability of the model. Validation of the NIR calibration model was performed according to International Conference on Harmonization (ICH) guidance Q2(R1) (ICH, 2005), and other regulatory guidance from the European Medicines Agency (EMA) (EMA, 2014) and the FDA (FDA, 2015), by assessing model linearity (expressed as correlation coefficient; R^2^), and accuracy (expressed as the absolute or relative error).

### 2.5 Thermal analysis

Differential scanning calorimetry (DSC) was used to characterise the powders and the drug-loaded printlets at 100 mm/s, 120 mm/s, 140 mm/s,160 mm/s and 180 mm/s laser scanning speeds. DSC measurements were performed with a Q2000 DSC (TA instruments, Waters, LLC, USA) at a heating rate of 10 °C/min starting from 45 to 300 °C. Calibration for cell constant and enthalpy was performed with indium (Tm = 156.6 °C, DHf = 28.71 J/g) according to the manufacturer instructions. Nitrogen was used as a purge gas with a flow rate of 50 mL/min for all the experiments. Data were collected with TA Advantage software for Q series (version 2.8.394), and analysed using TA Instruments.

For thermogravimetric (TGA) analysis, samples (average weight: 3–5 mg) were heated at 10 °C/min starting from 30 to 400 °C in open aluminium pans with a Discovery TGA (TA instruments, Waters, LLC, USA). Nitrogen was used as a purge gas with a flow rate of 25 mL/min. Data were collected and analysed by using TA Instruments Trios software. The results from thermal analysis were plotted using MATLAB software version R2019a (The MathWorks, CA, USA).

### 2.6 X-ray powder diffraction (XRPD)

The pure drugs, physical mixtures of drug and excipients, and 3D printed discs of 23 mm diameter × 1 mm height were analysed. The XRPD diffraction patterns were obtained in a Rigaku MiniFlex 600 (Rigaku, USA) using a Cu Kα X-ray source (λ = 1.5418 Å). The intensity and voltage applied were 15 mA and 40 k*V*. The angular range of data acquisition was 3–40° 2θ, with a stepwise size of 0.02° at a speed of 5°/ min.

### 2.7 Physical characteristics

#### 2.7.1 Printlet morphology

The diameter and thickness of the printlets were measured using a digital caliper (*n* = 3). Pictures were taken with an iPhone 7 camera.

#### 2.7.2 Breaking force

The crushing strength of each speed (n = 3) was measured using a traditional tablet hardness tester TBH 200 (Erweka GmbH, Heusenstamm, Germany), whereby an increasing force is applied perpendicular to the printlet axis to opposite sides of a printlet until the printlet fractures.

#### 2.7.3 Weight variation

Printlets of each laser scanning speed were weighed using weighing boat and calibrated balance. The average and standard deviation for the printlets were calculated (n = 3).

#### 2.7.4 Printlet density

Each printlet was measured for their height and diameter at 3 different points where the average was taken. The theoretical volume of the printlets was calculated using Eq. (1), to describe of volume of cylinder:

(1) $$V=\pi r^{2}*h$$

where V = volume, r = radius and h = height.

Density was then calculated using Eq. (2):

(2) $$\rho =\frac{m}{V}$$

where *ρ* = density, m = mass and V = volume.

### 2.8 Scanning electron microscopy

Surface images of the printlets were taken with a scanning electron microscope (SEM, JSM-840A Scanning Microscope, JEOL GmbH, Germany). All samples for SEM testing were coated with carbon (30–40 nm).

### 2.9 X-ray micro computed tomography (Micro-CT) imaging

A high-resolution X-ray micro computed tomography scanner (SkyScan1172, Bruker-microCT, Belgium) was used to 3D visualise the internal structure, density and porosity of the printlets. All oral formulations were scanned with a resolution of 2000 × 1048 pixels. 3D imaging was performed by rotating the object through 180° with steps of 0.4° and 4 images were recorded for each of those. The total acquisition time was 25 min per sample. Image reconstruction was performed using NRecon software (version 1.7.0.4, Bruker-microCT). 3D model rendering and viewing were performed using the associate program CT-Volume (CTVol version 2.3.2.0) software. The collected data was analysed using the software CT Analyzer (CTan version 1.16.4.1). Different colours were used to indicate the density of the printlets. Porosity values were calculated using the 3D analysis in the morphometry preview (200 layers were selected at the central part of the printlet as area of interest and analysed).

### 2.10 Determination of drug content

Individual printlets of each formulation were placed in separate volumetric flasks with deionised water (1000 mL). To each printlet, 12 drops of 5 M NaOH were added to each flask to increase the pH in order to dissolve the polymers under magnetic stirring until complete dissolution. A calibration model was developed across 0 – 50 mg/L and using Cary 100 UV–vis spectrophotometer (Agilent Technologies, UK) operated at 272 nm wavelength for quantification. 1 mL of samples of solution were removed and filtered through 0.22 μm filters (Millipore Ltd., Ireland) and then samples were put in 1 cm quartz cuvette for UV scanning.

### 2.11 Dissolution testing conditions

Drug dissolution profiles for the formulations were obtained with a USP-II apparatus (Model PTWS, Pharmatest, Germany) and involved the following: 1) The formulations were placed in 750 mL of 0.1 M HCl for 2 h to simulate gastric residence time, and then 2) 250 mL of trisodium phosphate (0.2 M) was added to adjust pH medium to 6.8 to mimic conditions of the small intestine. The paddle speed of the USP-II was fixed at 50 rpm and the tests were conducted at 37 ±0.5 °C. During the dissolution test, samples were automatically removed and filtered through 0.1 mm filters and drug concentration was determined using an in-line UV spectrophotometer (Cecil 2020, Cecil Instruments Ltd., Cambridge, UK) operated at 272 nm. Data were processed using Icalis software (Icalis Data Systems Ltd., Berkshire, UK).

The model developed by Moore and Flanner (Moore, 1996) was used to compare the dissolution profiles of devices of actual and predicted drug release profiles using an ƒ_2_ similarity test. The similarity factor (ƒ_2_) is a logarithmic reciprocal square root transformation of the sum of the squared error and can be calculated using the following Eq. (3):

(3) $$f_{2}=50\times \log\left\{{\left[1+\frac{1}{n}\sum_{t=1}^{n}{\left(R_{t}-T_{t}\right)}^{2}\right]}^{-\frac{1}{2}}\times 100\right\}$$

Where n is the number of dissolution time points, R_t_ and T_t_ are the percentage of drug released from the reference and test formulations at time point t, respectively (Gohel et al., 2009). The ƒ_2_ value ranges between 0 and 100 and a higher ƒ_2_ value indicates more similarity between the release profiles of the reference and test formulations (Shah et al., 1998).

## 3 Results and discussion

### 3.1 Development of 3D printed formulations

**Fig. 1:**
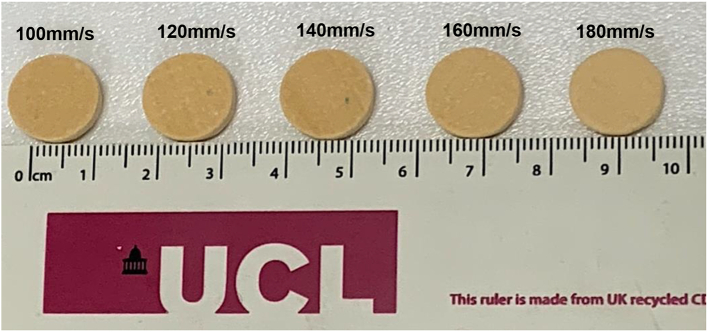
Images of cylindrical printlets from speeds 100–180 mm/s (left to right).

In this study, personalised printlets containing a narrow therapeutic index drug (10% *w/w* theophylline) were successfully produced using SLS 3D printing across a range of different laser speeds (100–180 mm/s) (Fig. 1). Speeds above the highest laser scanning speed of 180 mm/s were found to be too friable for handling. As the laser scanning speed increased, drug products were found to have a reduced colour intensity which was hypothesised to be due to a reduction in energy transfer to the materials used and hence a reduced sintering effect (Shirazi et al., 2015a).

**Fig. 2:**
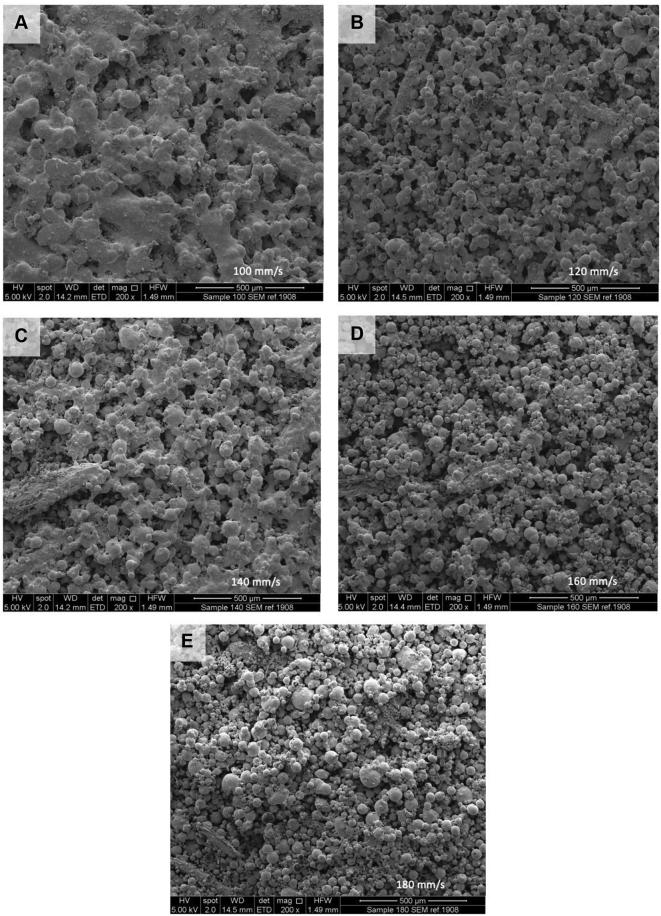
SEM images of the surface of printlets at speeds 100 mm/s (A), 120 mm/s (B), 140 mm/s (C), 160 mm/s (D) and 180 mm/s (E).

SEM and X-ray Micro CT imaging were conducted to further visualise the effects of laser speed on the drug product surface and internal structure. The printlets fabricated at the slowest laser scanning speed (100 mm/s; Fig. 2A) showed a higher degree of molten sintering on the surface compared to those at 180 mm/s, where single powder particles are easily identified in the structures (Fig. 2E). Upon increasing laser speed, an increased number of voids were found to be present on the tablet surface which was attributed to a reduced powder sintering effect.

**Fig. 3:**
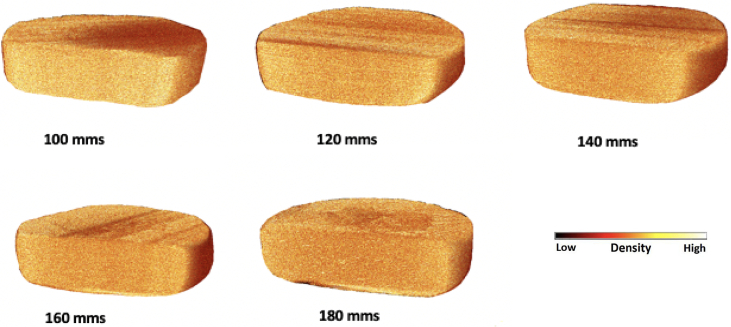
X-ray micro-CT images on cross-sections of 3D printlets.

**Fig. 4:**
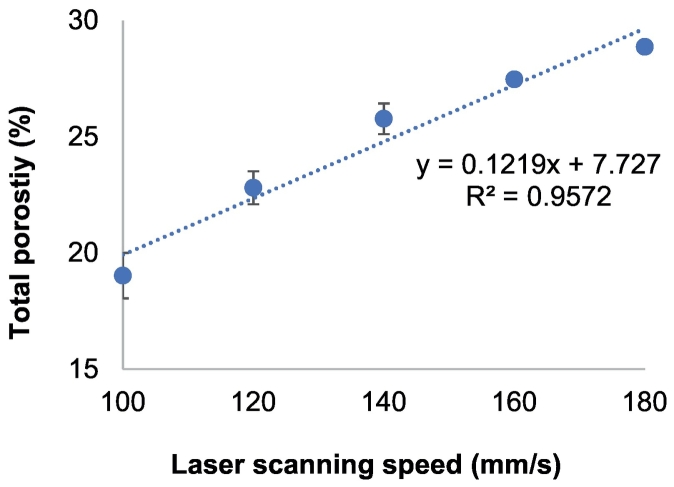
Printlets total porosity calculated using X-ray Micro CT imaging.

These findings were further confirmed using X-ray Micro-CT imaging, which showed that the slower laser scanning speeds produced drug products had an increased packing and less porous regions across the entire drug product matrix (Fig. 3). The total porosity of the printlets was also calculated which is shown in Fig. 4. For the purpose of this study, porosity refers to the empty spaces in printlets and is a fraction of the volume of voids over the total volume as a percentage between 0 and 100%. There was found to be an increase in the porosity of printlets produced at 180 mm/s (28.87%) when compared with the 100 mm/s printlets (19.02%).

This can be explained by the effect of laser sintering energy on the powder particles: during the laser application to the powder bed, the local temperature is increased (Gao et al., 2008). Consolidation of amorphous polymers (as in this case of Eudragit L100–55) occurs when the powder particles are heated to at or above the glass transition temperature (T_g_), whereby a rapid decrease in elastic modulus (*E*) occurs (Shirazi et al., 2015a). The T_g_ of Eudragit L100–55 has previously been reported to be 110 °C (Thakral et al., 2013), which was matched with the surface temperature of the SLS printing process to ensure a temperature just beyond the T_g_ was met. At slower laser scanning speeds, more dense and less porous drug products were produced due to a longer interaction time between the powder and the laser beam, leading to a higher transmission of energy to the powder bed (Shirazi et al., 2015b). As such, a higher degree of polymer melting occurs, enabling a higher proportion of liquid phase to flow and infiltrate into the voids and the formation of liquid-solid bridges between powder particles, in turn leading to a denser construct which has been demonstrated elsewhere (Fred et al., 2014). Conversely, a higher laser scanning speed results in less energy transferred to the materials, leading to less sintering and in turn to increased porosity (Shirazi et al., 2015a). Such an effect was forecast to impact the critical quality attributes (CQAs) of the printlets, such as hardness, printlet weight and drug release.

**Fig. 5:**
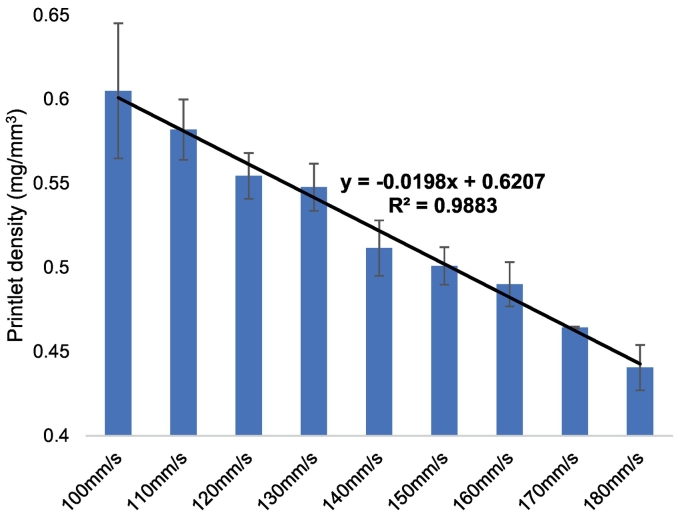
Average calculated density of printlets of speeds 100–180 mm/s with a decreasing trend.

The impact of varying scanning speeds on the physical characteristics (drug product weight, density and hardness) of the printlets were analysed (Table 2). In general, it was found that a slower laser scanning speed correlated with an increase in the weight and density of the printlets compared with the faster speeds. In particular, a linear correlation was observed between laser scanning speed and printlet density (R^2^ = 0.9883, Fig. 5). Interestingly, this finding will have a direct impact on the dosage which is formulated and administered to a patient; for example, the 100 mm/s laser scanning speed would deliver a dosage of ∼33 mg, compared with only ∼19 mg for the 180 mm/s formulation. As such, for SLS 3D printing technologies to be used clinically, it would be of paramount importance to determine the most appropriate laser scanning speed in relation to the intended dosage that is required to be administered.

The laser scanning speed was also found to affect the breaking force of the printlets, with the slower laser scanning speeds exhibiting a higher breaking force compared with the faster speeds (Table 1). As an example, the mean breaking force exceeded 485 N for the 100 mm/s speeds, compared with 16 N for the 180 mm/s speed. In general, the recommended crushing strength for tablets is >40 N (Ayorinde, 2012) and, as such, the majority of laser scanning speeds were found to pass this requirement.

**Table 1:** Physical characteristics of printlets.

Scanning speed (mm/s)	Weight (mg ± SD)	Height (mm ± SD)	Breaking force (N ± SD)	UV Recovery (% ± SD)
100	334.5 ± 22.7	3.56 ± 0.28	>485	100.70 ± 0.98
110	259.7 ± 5.40	3.61 ± 0.18	353.7 ± 38.28	107.29 ± 0.48
120	291.0 ± 25.1	3.60 ± 0.27	235.3 ± 9.61	94.00 ± 1.20
130	272.8 ± 24.1	3.81 ± 0.14	197.0 ± 23.07	105.04 ± 0.92
140	275.0 ± 2.90	3.68 ± 0.01	110.0 ± 20.30	115.06 ± 0.92
150	255.3 ± 20.4	3.65 ± 0.05	95.7 ± 4.04	93.56 ± 1.43
160	225.7 ± 13.8	3.50 ± 0.12	88.0 ± 14.42	101.01 ± 0.52
170	205.0 ± 18.7	3.41 ± 0.14	58.7 ± 10.69	114.17 ± 0.77
180	187.8 ± 9.08	3.39 ± 0.22	16.0 ± 00.00	102.03 ± 2.28

Drug content analysis showed no drug degradation during the printing process as the values were similar to the theoretical drug loading (10% *w/w*) (Table 1). Values were found to be within the acceptable limits of the British Pharmacopeia, which are between 85 and 115% (Uddin et al., 2015). This shows the versatility of SLS printing to produce medicines with different laser speeds and also allows the further application of SLS 3DP technology to narrow therapeutic index (NTI) drugs in the pharmaceutical field.

### 3.2 Solid state analysis

**Fig. 6:**
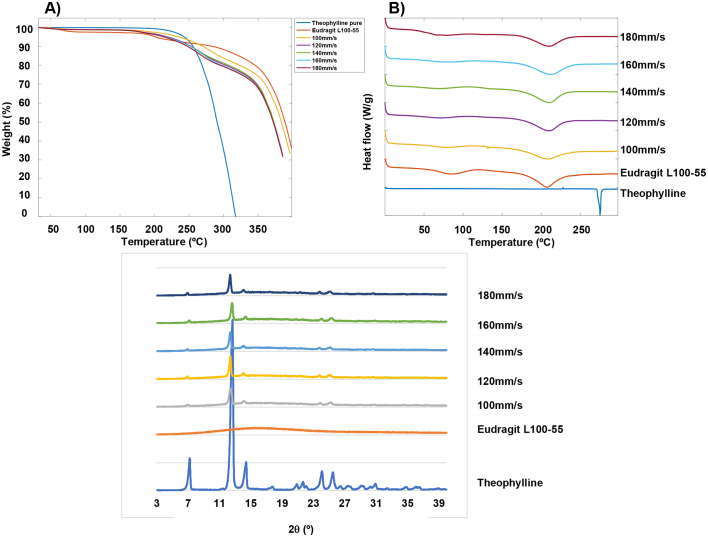
Solid state characterisation of pure theophylline, Eudragit L100–55 and theophylline 10% unsintered formulation blends and printlets of speeds 100–180 mm/s; (A) Thermogravimetric analysis; (B) DSC thermographs and; (C) X-ray powder diffractograms.

Thermal analyses were performed on all the pure ingredients, unsintered formulation blends and sintered tablets to identify the solid-state characteristics of the drug within the polymer matrix. TGA analysis showed that Eudragit L100–55 lost approximately 1.8% of its weight after reaching 100 °C due to the release of surface water evaporation (Fig. 6A). The second mass loss of ∼3% at 200 °C is associated with the onset of degradation (previously identified in the literature as ∼176 °C (Sawant et al., 2018)). Theophylline onset of degradation was found to occur at around 220 °C which is similar to what has been reported in the literature (Pietrzak et al., 2015). Favourably, the SLS printing temperature used in this study (110 °C) is well below the degradation point of the drug and excipients, thereby indicating its suitability for the production of printlets. This was confirmed via the observation of a similar TGA patterns between the printlets and the pure ingredients (Fig. 6A).

DSC analysis (Fig. 6B) showed that pure theophylline exhibited a melting endotherm at approximately 273 °C, which indicates the drug crystallinity. Eudragit L100–55 exhibited two very broad melting endothermic peaks (at around 75 °C and 210 °C). The first was due to side chain mobility (β-relaxation) and the second was due to the anhydride formation resulting from water evaporation during the heating process of the DSC scan. In addition, the glass transition temperature (T_g_) which appeared at ∼120 °C represented the main polymeric linear chain mobility (α-relaxation). The inclusion of Eudragit L100–55 in the printlets of all speeds (100–180 mm/s) shows an absence of the characteristic theophylline melting endotherm at 273 °C.

XRPD diffractograms (Fig. 6C) of the drug-polymer mixture and printlets show diffraction peaks at 2θ = 7°, 12°, 14°, 24° and 25° which matches the diffraction pattern of crystalline theophylline (Räsänen et al., 2001). As expected, Eudragit L100–55 was found to be fully amorphous indicated by the absence of Bragg diffraction peaks and broad halo in the XRPD diffractogram. In contrast to the DSC thermographs, the XRPD diffractograms showed for the 3D printed formulations that theophylline was found to be partially retained in the crystalline form. Previous studies that have 3D printed theophylline also found a full or partial drug crystallinity present post-printing, likely due to the high melting point of theophylline (273 °C) which is not reached during the 3D printing processes (Giri et al., 2020; Isreb et al., 2019; Okwuosa et al., 2017; Okwuosa et al., 2016). The differences between the XRPD and DSC findings may be due to the inherent differences between the two analytical techniques, whereby XRPD analyses only the surface of the formulation at room temperature which, in previous research, has been found to retain unsintered material on the surface in the pores of the SLS 3D printed formulation. The absence of melting endotherms within the DSC analysis may be due to the molecular dispersion of theophylline within the Eudragit L100–55 polymer matrix as the termpature increased during the DSC procedure, leading to reduced crystallinity that could not be detected with DSC. A similar trend was found in other studies with theophylline and HPC (Giri et al., 2020).

### 3.3 Drug dissolution

**Fig. 7:**
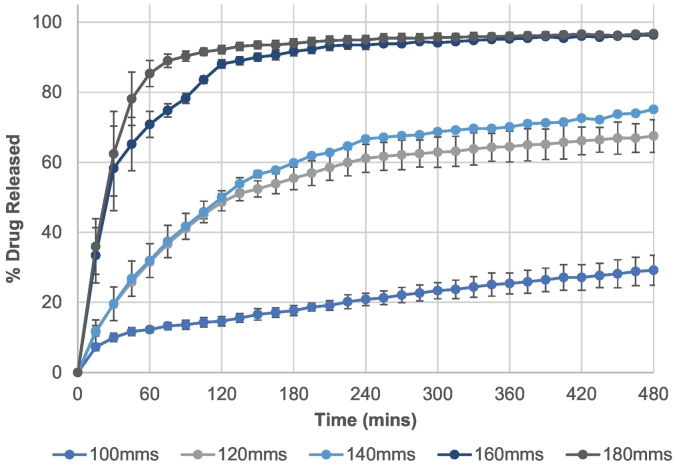
Drug dissolution profiles of printlets fabricated at speeds 100 m/s, 120 mm/s, 140 mm/s, 160 mm/s and 180 mm/s over 8 h.

Using the USP II dissolution in vitro model, all printlets were tested for their drug release characteristics across 8 h (Fig. 7). The dissolution model is designed to mimic both the gastric and intestinal conditions of the gastrointestinal tract by dissolving the printlets in acidic medium (pH 1.2; gastric phase) for the first 120 mins before adjusting the pH of medium to pH 6.8 (intestinal phase) by adding phosphate buffer.

The rate of drug release was found to increase as a function of laser scanning speed. As an example, at 240 mins, printlets fabricated at 180 mm/s reached 95% drug release whereas printlets at 140 mm/s and 100 mm/s reached only 67% and 21%, respectively. These values can be explained by the physical characteristics of the formulations where at a higher laser speed, a more porous and less dense printlet is produced. In turn, due to an increased number of pores, this allows more media to come into contact with printlet, leading to faster dissolution rate. This effect can also be observed in the general trend of the drug release profiles; with printlets produced using the faster laser scanning speeds, a burst effect of drug release is seen initially; i.e., within the first 15 mins for speeds 160 mm/s and 180 mm/s, around 30–35% of drug is released. In comparison, for speeds 100–140 mm/s, only 7–11% is released. This is likely due to the more porous printlets allowing an increased influx of water into the matrix formulation, enabling the formation of microchannels to facilitate rapid dissolution.

For the most dense printlet (100 mm/s), drug release profiles that are similar to a sustained release profile are observed (a gradual increase in drug release over time) which may be due to an erosion-mediated disintegration mechanism (Goyanes et al., 2016). Despite the use of Eudragit L100–55, a polymer designed to dissolve and release drug >pH 5.5 (in the small intestinal compartments), theophylline was released during the first 120 mins (acidic medium) for all formulations, likely due to the drug being present within the pores on the surface of the drug product (Fig. 6C).

### 3.4 NIR spectroscopic measurements

Previous studies have highlighted the potential for non-destructive NIR spectroscopic methods to be able to predict the physical characteristics of tablets, including density and porosity (Donoso et al., 2003; Otsuka, 2006), hardness (Kandpal et al., 2017; Otsuka and Yamane, 2006; Qiushi et al., 2019) and drug release (Ojala et al., 2020) due to the sensitivity to surface and internal structural effects. As an example, reflectance NIR spectroscopy has previously been used to quantify drug release in tablets manufactured at different compression levels (Baranwal et al., 2019). The researchers found that denser dosage forms were produced upon increasing compression level, which in turn increased the amount of NIR absorbance due to a reduced scattering effect.

Conversely, to date, no studies have evaluated the use of PAT technologies to predict the density and drug release of printed pharmaceuticals. To that end, the evaluation of reflectance FT-NIR spectroscopy combined with both univariate analysis was carried out in order to predict drug product density and drug release at 2 h and 4 h timepoints, with the aim to provide a non-destructive and all-in-one characterisation of the physical properties of printlets.

**Fig. 8:**
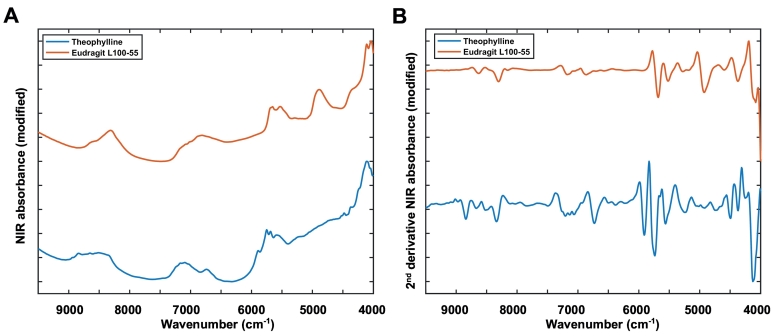
Raw (A) and 2nd derivative FT-NIR absorbance spectra (B) of pure ingredients; theophylline and Eudragit L100–55.

Initially, the pure ingredients (theophylline and Eudragit L100–55) were scanned using a portable reflectance FT-NIR spectroscopy set up (Fig. 8). The polymer absorbance at 5800–6000 cm^−1^ and 4200–4500 cm^−1^ was found to correspond to the CH–CH first overtone and combination bands, respectively (Figs. 8A and B) (Tabasi et al., 2009). The NIR spectra of theophylline anhydrous were mainly due to -C-H stretching bands of the methine carbons (5900–6100 cm^−1^) and combination bands due to the -N-H stretching vibrations (4000–4500 cm^−1^) (Korang-Yeboah et al., 2016).

**Fig. 9:**
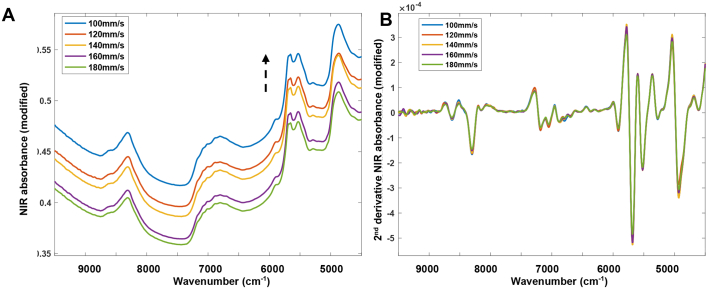
Overlaid A) Raw and B) 2nd derivative FT-NIR spectra of printlets produced at 100 mm/s, 120 mm/s, 140 mm/s, 160 mm/s and 180 mm/s measured in the reflectance mode.

The reflectance NIR absorbance spectra of printlets produced at laser speeds of 100 mm/s, 120 mm/s, 140 mm/s, 160 mm/s and 180 mm/s were evaluated (Fig. 9A). Selection of the most appropriate data pre-processing method is important for getting a robust calibration curve as the unique attribute of NIR spectroscopy is that the spectra are dependent upon both the chemical composition of the sample and the physical properties of the sample.

For tablets, the physical attributes often show up as a shift in the baseline, as demonstrated elsewhere (Tanabe et al., 2007). It can be seen that for the raw data, as the laser speed decreases and hence density of the drug product increases, an increasing baseline shift occurs and NIR absorbance occurs (Fig. 9A). This effect is likely due to the difference in surface presentation, whereby lower porosity printlets (produced at slower scanning speeds) exhibit a smoother surface, thereby causing less diffuse reflectance and higher absorbance compared with the higher porosity printlets (Otsuka et al., 2007; Tsuchikawa and Tsutsumi, 2002). In contrast, when converting the data into the 2nd derivative, this effect was diminished and there was no apparent correlation between laser scanning speed and NIR absorbance (Fig. 9B). Previous studies have highlighted that using the second derivative to quantify physical attributes such as tablet crushing strength and dissolution rate is counterproductive, as this pre-processing method aims to reduce information related to physical attributes (such as particle size effects, or scattering effects) (Tatavarti et al., 2005). As such, the raw spectra were used throughout future model development.

**Fig. 10:**
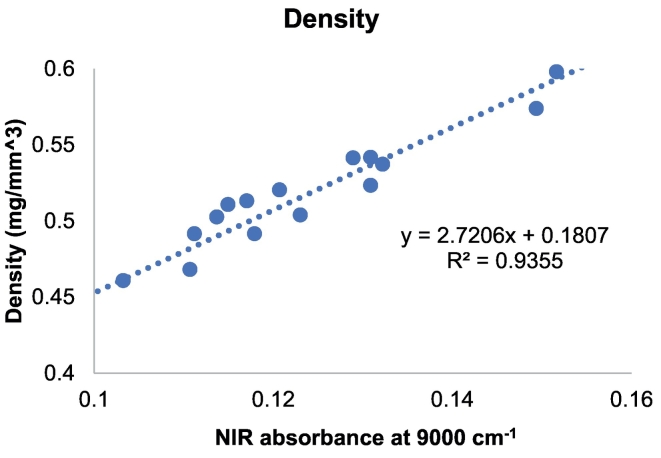
Univariate calibration model of NIR absorbance vs. actual printlet density (mg/mm^3^).

For the prediction of density, a univariate calibration model was developed by plotting the NIR absorbance at 9000 cm^−1^ against the actual density measurements of the 3D printed drug products. (Fig. 10). The absorbance at 9000 cm^−1^ was selected due to it exhibiting the excellent linearity (> 0.9 R^2^) between absorbance and the drug product physical properties. The developed calibration model included all laser scanning speeds, with 5 tablets selected as the test set to evaluate the predictive ability of the model. A positive linear correlation (R^2^ = 0.9355; Fig. 10) was found between NIR absorbance and density and the model was able to accurately predict density values with a low error (<0.026 mg/mm^3^; Table 2).

**Table 2:** Actual vs. predicted density for test printlets.

Laser scanning speed (mm/s)	Density actual (mg/mm^3^)	Density predicted (mg/mm^3^)	Absolute Error (mg/mm^3^)
100	0.5676	0.5932	−0.026
120	0.5419	0.5193	0.023
140	0.5175	0.5041	0.013
160	0.4682	0.4880	−0.020
180	0.4541	0.4742	−0.020

**Fig. 11:**
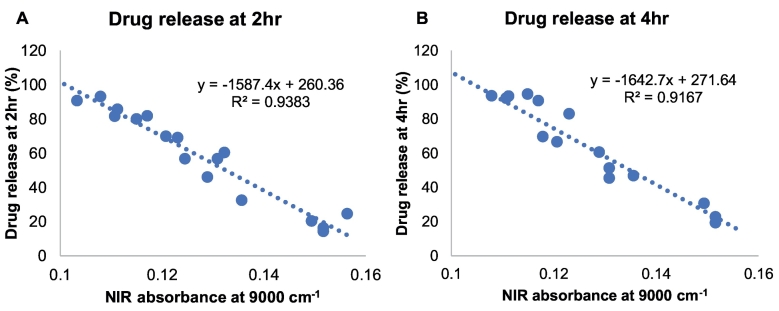
Univariate calibration models of NIR absorbance at 9000 cm^−1^ vs. actual printlet drug release (%) at (A) 2 h and (B) 4 h.

Univariate calibration models were also explored for drug release at 2 h and 4 h. In a similar manner to the density calibration model, all laser scanning speeds were included, with 5 tablets selected as the test set to evaluate the predictive ability of the model. A linear correlation was found between NIR absorbance at the 9000 cm^−1^ and drug release in both cases (R^2^ = 0.9383 and 0.9167 for 2 h and 4 h, respectively; Figs. 11A and B). In general, a good predictive performance was observed between the actual and predicted drug release values with a low error (< 4.4%; Table 3, Table 4).

**Table 3:** Actual vs. predicted drug release at 2 h for test printlets.

Laser scanning speed (mm/s)	Actual drug release at 2 h (%)	Predicted drug release at 2 h (%)	Error (%)
100	15.36	13.50	1.86
120	56.95	52.62	4.33
140	69.94	71.68	−1.74
160	81.65	77.90	3.75
180	92.33	89.14	3.20

**Table 4:** Actual vs. predicted drug release at 4 h for test printlets.

Laser scanning speed (mm/s)	Actual drug release at 2 h (%)	Predicted drug release at 2 h (%)	Error (%)
100	20.36	16.18	4.18
120	76.38	73.09	3.29
140	69.82	76.39	−6.57
160	92.24	89.79	2.45
180	94.78	94.45	0.33

**Fig. 12:**
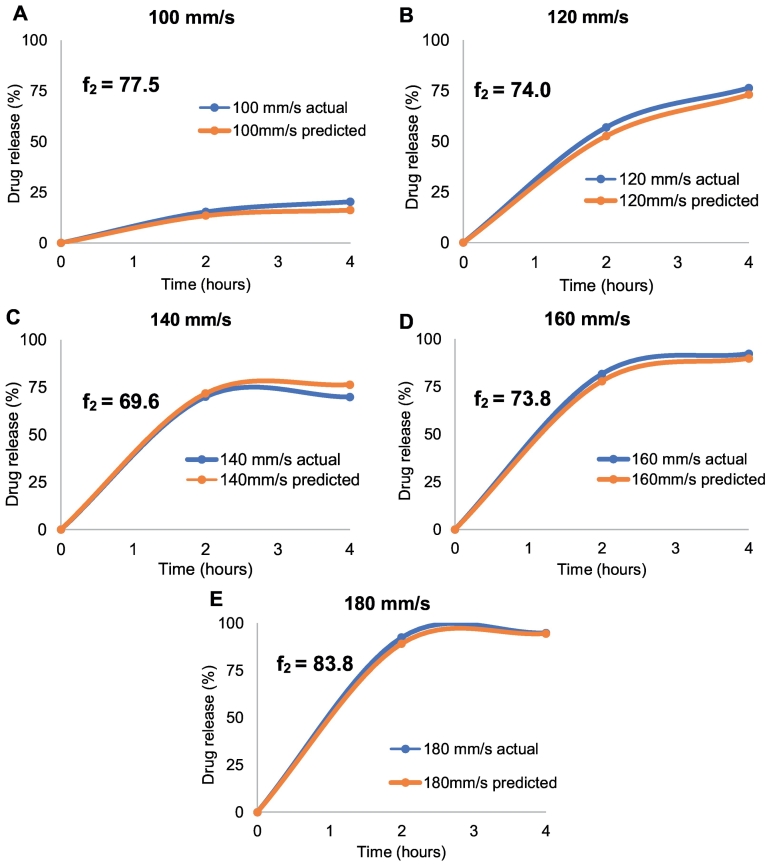
Actual vs. predicted dissolution profiles for five printlets of A) 100 mm/s, B) 120 mm/s, C) 140 mm/s, D) 160 mm/s and E) 180 mm/s. All printlets were considered statistically similar as per f_2_ analysis.

For each of the test sets (individual samples of 100 mm/s, 120 mm/s, 140 mm/s, 160 mm/s and 180 mm/s printlets), the actual vs. NIR predicted drug release were overlaid for comparison (Fig. 12A-E). Statistical analyses were performed by way of the similarity factor (f_2_) to compare the similarities between the actual vs. predicted dissolution profiles for each of the speeds. Favourably, all of the printlets had an f_2_ value that exceeded 50, suggesting that the dissolution profiles were statistically similar.

There results demonstrate a proof-of-concept that NIR spectroscopy could be used for density and drug dissolution prediction. It is worth noting that this study focussed on only one drug and polymer combination and, in future work, the model would require validation or further development (e.g., including different drug-polymer combination variations) before application to other drug product types. It is also worth noting that these results were based on a test set of only *n* = 5 and at 3 time points (0 h, 2 h and 4 h). To ensure the confidence in the results and to be acceptable in terms of regulatory requirements, an increased number of test sets and timepoints are required to be analysed in the future.

## 4 Conclusion

Overall, this study has demonstrated the ability of SLS 3D printing to create drug products loaded with a narrow therapeutic index drug (theophylline) that exhibit different drug release profiles upon modulation of the laser scanning speed. The printed drug products formulated with a higher laser speed exhibited an accelerated drug release compared with the slower laser scanning speeds. This study has also demonstrated the feasibility of using reflectance FT-NIR spectroscopy as a non-destructive approach to predict printlet density and drug release at 2 h and 4 h. Univariate calibration models were developed based on a baseline shift in the spectra in the third overtone region upon changing physical properties. For density prediction, the developed univariate model had high linearity (R^2^ value = 0.9335) and accuracy (error < 0.026 mg/mm^3^). For drug release prediction at 2 h and 4 h, the developed univariate models demonstrated a linear correlation (R^2^ values of 0.9383 and 0.9167, respectively) and accuracy (error < 4.4%). The predicted vs. actual dissolution profiles were found to be statistically similar (f_2_ > 50) for all of the test printlets. The results presented here show the potential of using NIR spectroscopy for the prediction of printlet physical properties including density and dissolution behaviour.

## Declaration of Competing Interest

The authors declare that they have no known competing financial interests or personal relationships that could have appeared to influence the work reported in this paper.

## Data Availability

Data will be made available on request.
